# Effect of social capital on enrolment of informal sector occupational groups in the national health insurance scheme in Ghana: a cross-sectional survey

**DOI:** 10.1186/s12913-024-11025-9

**Published:** 2024-04-29

**Authors:** Eric Nsiah-Boateng, Patricia Akweongo, Justice Nonvignon, Moses Aikins

**Affiliations:** 1 Department of Health Policy, Planning and Management, School of Public Health, University of Ghana, Legon, Accra, Ghana; 2 Research, Policy, Monitoring and Evaluation Directorate, National Health Insurance Authority, Accra, Ghana; 3 Policy, Planning, Monitoring and Evaluation Directorate, Ministry of Health, Accra, Ghana

**Keywords:** Social capital, Health insurance enrolment, Informal sector workers, National health insurance scheme, Ghana

## Abstract

**Background:**

Enrolment of informal sector workers in Ghana’s National Health Insurance Scheme (NHIS) is critical to achieving increased risk-pooling and attainment of Universal Health Coverage. However, the NHIS has struggled over the years to improve enrolment of this subpopulation. This study analysed effect of social capital on enrolment of informal sector workers in the NHIS.

**Methods:**

A cross-sectional survey was conducted among 528 members of hairdressers and beauticians, farmers, and commercial road transport drivers’ groups. Descriptive statistics, principal component analysis, and multinomial logit regression model were used to analyse the data.

**Results:**

Social capital including membership in occupational group, trust, and collective action were significantly associated with enrolment in the NHIS, overall. Other factors such as household size, education, ethnicity, and usual source of health care were, however, correlated with both enrolment and dropout. Notwithstanding these factors, the chance of enrolling in the NHIS and staying active was 44.6% higher for the hairdressers and beauticians; the probability of dropping out of the scheme was 62.9% higher for the farmers; and the chance of never enrolling in the scheme was 22.3% higher for the commercial road transport drivers.

**Conclusions:**

Social capital particularly collective action and predominantly female occupational groups are key determinants of informal sector workers’ participation in the NHIS. Policy interventions to improve enrolment of this subpopulation should consider group enrolment, targeting female dominated informal sector occupational groups. Further studies should consider inclusion of mediating and moderating variables to provide a clearer picture of the relationship between occupational group social capital and enrolment in health insurance schemes.

**Supplementary Information:**

The online version contains supplementary material available at 10.1186/s12913-024-11025-9.

## Background

A growing number of the world’s population do not have financial risk protection for health care. Therefore, they pay out-of-pocket (OOP) in an event of ill-health, which often pushes them into poverty if the cost of care constitutes a substantial proportion of their household expenditure [1]. Those who forgo care because they could not afford the cost end up having poor health. Efforts to addressing this challenge in low-and middle-income countries (LMICs) and move towards Universal Health Coverage (UHC) have led to implementation of different forms of health insurance schemes in the last two decades. Population coverage of these prepayment schemes in Sub-Saharan Africa (SSA), however, remains proportionally low, ranging between 3 and 60% [2–6] except for a community-based health insurance scheme (CBHI) for the informal sector workers in Rwanda, which reportedly has 80% coverage [7].

Ghana has made progress in enrolment in its National Health Insurance Scheme (NHIS), provision of financial access to health care, and contribution to healthcare providers’ financial resources [8–10]. Nonetheless, the proportion of informal sector workers in the scheme has historically remained low, constituting a little over one-third of the total enrolees in each year [11, 12]. This situation is a major concern to managers (National Health Insurance Authority (NHIA) and other stakeholders of the scheme given that about 80% of the economic workforce is in the informal sector economy [13]. In addition, this subpopulation is the only group that pays premium directly to the scheme; thus, their consistent low enrolment has the tendency to affect risk-pooling, revenue albeit low, and ultimately the overarching goal of achieving UHC by 2030. At the individual level, the uninsured informal sector worker may have limited access to healthcare services and likely to be pushed into poverty in an event of catastrophic payment for healthcare.

Literature shows that social capital, operationalised in this study as “features of social organisation such as networks, norms, and social trust that facilitate coordination and cooperation for mutual benefit” [14] is associated with participation of occupational groups in social protection programmes. Social capital factors, including membership in groups and networks facilitate collective decision-making such as participation in social protection programmes [15, 16]. Occupational groups that are less diverse (or more homogenous) would trust and cooperate with each other to undertake collective actions for the development of their wellbeing. Similarly, groups with members who are well-connected to friends and family and who help each other in times of need, receive financial support towards their wellbeing [6, 17]. This mutual support helps occupational group members to contribute money to participate in social protection programmes or support activities of the group that may not benefit them directly. Other occupational group social capital factors such as members’ perception of trust, including trust in their occupational groups; trust in state agencies; and trust in their family, friends and community influence their decision to participate in social health insurance programmes [6, 18]. Besides, members’ adherence to occupational group norms such as sharing and reciprocity, facilitate collective decision to participate in social protection programmes. It is also evident in literature that occupational groups whose members trust each other and support each other in emergency situations are more willing to participate in collective decision-making towards their wellbeing [16].

Earlier studies have provided reasons for the low enrolment of informal sector workers in health insurance programmes in LMICs and suggested recommendations to improve the situation [4, 5, 19, 20]. These studies, however, provide little evidence to explain the influence of social capital such as membership in occupational groups, trust, and collective action on enrolment of informal sector occupational groups in health insurance programmes, a gap this study sought to fill. A comprehensive application of these three dimensions of social capital and their effect on national level health insurance enrolment is very limited in literature. One study that employed the concept of social capital to examine its effect on NHIS enrolment used community and institutional trust as a proxy for measuring social capital [18], as opposed to the increasing suggestion in literature for inclusion of membership in groups and networks (structural social capital) as a variable in the assessment of social capital [16]. The authors also measured social capital at the individual level, contrary to the ecological level recommended in literature [21, 22]. Our study therefore sought to examine the effect of informal sector occupational group social capital as a collective attribute on enrolment in the NHIS.

## Methods

### Study design and setting

A cross-sectional study was conducted between December 2018 and February 2019 in selected districts of four administrative regions across the three ecological zones (southern, middle, and northern) of the country. Key demographic and socio-economic variables for the study area [23–29] are summarised in Supplementary Table 1.

## Study population

The study population comprised five informal sector occupational groups. These groups were the private commercial road transport drivers in the Achimota transport terminal in the Greater Accra region; hairdressers and beauticians association in the Greater Accra region; community-based Cocoa farmer group (Kokoo Pa Association) in Ashanti and Ahafo regions; and two maize and soya bean farmer associations in the Upper East region. The private commercial road transport drivers are mainly males; the hairdressers and beautifications are predominantly females; the cocoa farmers are predominantly males; and the maize and soya bean farmers are only females.

## Sampling method

We identified the informal sector occupational groups through the Ghana Trade Union Congress Office and friends. We visited these occupational groups and interacted with them for possible inclusion in the study. Inclusion of occupational groups in the study was based on: 1) the three occupational sectors of the economy such as agriculture, service and industry; 2) membership ties and activeness of the group, measured by frequency of meetings and payment of membership dues over the last three years (2015–2017), as evidence shows that such groups perform better than groups with fewer ties [30, 31]; 3) decentralised communication pattern; and 4) high member heterogeneity, density, and whole-network centrality [32–34].

We then obtained list of names of each selected occupation group from the leaders and sampled within them using a simple random sampling design [35, 36]. Cochran’s sample size formula for categorical data or a research where categorical variable plays a major role in the data analysis, Eq. (1); and the correction formula for situations where the required sample size exceeds 5% of the population, Eq. (2), was used to determine the required sample size for each occupational group. These group-specific sample sizes were summed up to obtain the total sample size for the study [37, 38].

1 $$\underline{\text{n}}_\text{o}=\frac{{\left(\underline{\text{t}}\right)}^2\ast\left(\text{p}\right)\left(\text{q}\right)}{{\left(\underline{\text{d}}\right)^2}}$$

2 $$\underline{\text{n}}_{1} = \frac{\underline{\text{n}}_{\text{o}}}{\left(1 +\underline{\text{n}}_{\text{o}}/{\text{Population}}\right)}$$

where t = value for selected alpha level of 0.025 in each tail [1.96]; (p)(q) = estimated variance [0.25] (for a maximum possible sample size); d = acceptable margin of error for proportion being estimated [0.05]; n_o_ = required return sample size according to Cochran’s formula [384]; and n_1_ = required return sample size because sample is greater than 5% of population.

A total of 481 individuals were sampled for the study; however, this was increased by 10% to cater for non-response rate [37]. Microsoft excel 2016 was used to compute the estimated sample size for each occupational group.

## Data analysis

Social capital was measured as a collective attribute of the group using proxy indicators of: 1) membership in groups and networks (diversity, democratic functioning, bonding, bridging); 2) trust and adherence to group norms (trust in group, trust in linguistic group, transaction trust, solidarity); and 3) collective action (sanctions for not participating in collective action, contribute time or money towards common development goal, cooperate to try to solve a problem, etc.) [16]. Respondents were asked to rate the questions for these proxy indicators on a Likert scale, ranging from 1 (disagree strongly) to 5 (agree strongly). Indices (ranged between 0 and 1) for the membership in groups and collective action proxy indicators were determined by dividing the sum of the individual scores of each question by the respective maximum sum of scores. The results were averaged over the occupational groups to obtain an ecological social capital mean for each group and assigned it to each member. This analytical approach has been validated in other studies [21, 22].

Trust was, however, analysed using Principal Component Analysis (PCA) to reduce the large number of hypothetical questions into smaller components or factors of uncorrelated variables, as applied in other social capital studies in LMICs [18, 22, 39] and high-income countries [40]. The PCA showed a Kaiser–Meyer–Olkin (KMO) Measure of Sampling Adequacy of 0.7601, and a significant Bartlett test of sphericity (*p* < 0.001), indicating that the 12 questions were intercorrelated; thus, warranted the use of PCA to reduce them to uncorrelated principal components. Four uncorrelated components with eigenvalue of 1 or more, which accounted for 65.6% of the variance in the data were extracted (Supplementary Table 2). The extracted components or factors and their respective proportions were: 1) trust in occupational and linguistic groups (30.3%); 2) solidarity (16.2%); 3) trust in NHIS and healthcare providers (9.7%); and 4) trust in transactions (9.4%). The rotated factor coefficients showed that five variables of trust and solidarity loaded heavily on factor 1; three on factor 2; and two each on factor 3 and 4 (Supplementary Table 3). The overall scale reliability coefficient for the 12 variables showed internal consistency in scale reliability (Cronbach’s α = 0.7526). Likewise, the respective Cronbach’s alpha for the four extracted components showed internal consistency in scale reliability (Cronbach’s α = 0.7494, 0.7792, 0.7619 and 0.2966), although the last principal component factor “trust in transactions” was far lower.

A multinomial logit regression model was then performed to determine effect of informal sector occupational group social capital on NHIS enrolment based on the assumption that the error term is logistically distributed (not normally distributed), and the errors are uncorrelated). This analytical technique has been employed in other studies to examine determinants of enrolment in the NHIS [18, 41, 42]. The outcome variable “enrolment status” was categorised into three unordered alternatives: currently enrolled (1); previously enrolled (2); and never enrolled (3). The base or reference category for comparison was “never enrolled (3)” and the main predictor or independent variables were the measures of social capital: diversity of membership; pattern of democratic functioning; bonding; bridging; trust in occupational and linguistic groups; trust in agencies (NHIS and healthcare providers (HCP)); solidarity; trust in specific transactions; and collective action. The control or other independent variables were the sociodemographic characteristics of the occupational group members (age, sex, marital status, education, etc.). The regression Eqs. (3) and (4) below were applied:

3 $$In\,\left(\frac{p\left(enrolment\,status=currently\,enrolled\right)}{P\left(enrolment\,status=never\,enrolled\right)}\right)\,=b10+ b11SC+b12X......$$

4 $$In\left(\frac{p\left(enrolment\,status=previously\,enrolled\right)}{P\left(enrolment\,status=never\,enrolled\right)}\right)=b20+b21SC+b22X......$$

where *b’s* are the regression coefficients; *SC* is a vector of occupational group social capital; and *X* is a vector of occupational group sociodemographic characteristics.

Prior to the estimation of multinomial regression, a correlation test was used to help select the appropriate variables for inclusion in the model. Covariates that showed collinearity at (*r* > 0.5) were excluded from the model to ensure a stable model [43]. All Nonetheless, factors found in literature to be associated with the outcome variable “enrolment status” were included in the model [5]. Threshold for statistical significance was set at* p* < 0.05 and results of the model presented in a table.

To understand the model better, we estimated the average marginal effects for the covariates for each outcome alternative (Supplementary Table 4). We also estimated predicted probabilities of choosing each NHIS enrolment status (currently enrolled, previously enrolled, never enrolled) for each occupational group, holding all other variables at their means. The predicted probabilities by occupational group were then plotted for each NHIS enrolment status.

Post-estimation tests such as Hosmer–Lemeshow goodness-of-fit test [44, 45] and STATA command “fitstat” were also performed to assess how the independent variables fit the model.

## Results

### Characteristics of the study participants

A total 495 (94%) occupational group members out of 528 sampled for the survey responded to the questionnaire, of which 215 (43.4%) were beauticians and hairdressers (Table 1). A total of 172 (34.7%) occupational group members were currently enrolled in the NHIS at the time of the survey. Regarding the health-related characteristics of the group, 242 (48.9%) rated their health status as very good; and 180 (53.3%) used hospital as their usual source of care. Average age of the survey participants was 43.7 years (*SD* = 11.51); 294 (59.4%) were females; 231 (46.7%) belonged to the Akan ethnic group; and 353 (71.3%) lived in urban centres. In addition, 375 (75.8%) of the survey participants were married or co-habiting, and the average household size was approximately 6 persons (*SD* = 3.18), with the farmers having the highest average of about 8 persons (*SD* = 3.72) per household. Moreover, 286 (57.9%) of the participants surveyed were middle school or Junior High School (JHS) graduates; 398 (80.4%) were self-employed; and the median^1^ monthly income was GH¢600.00 (US$115.16^2^).

**Table 1:** Descriptive statistics of study participants

Variable	Farmers	Commercial road transport drivers	Beauticians and hairdressers	All
	n (%)	n (%)	n (%)	n (%)
**Overall**	130 (100)	150 (100)	215 (100)	495 (100)
**NHIS enrolment status**				
Currently enrolled	51 (39.2)	17 (11.4)	104 (48.4)	172 (34.7)
Previously enrolled	60 (46.2)	74 (49.3)	91(42.3)	225 (45.5)
Never enrolled	19 (14.6)	59 (39.3)	20 (9.3)	98 (19.8)
**Self-reported health status**				
Very good	56 (43.1)	81 (54.0)	105 (48.8)	242 (48.9)
Good	38 (29.2)	46 (30.7)	64 (29.8)	148 (29.9)
Fair	28 (21.5)	20 (13.3)	43 (20.0)	91 (18.4)
Poor	8 (6.2)	3 (2.0)	3 (1.4)	14 (2.8)
**Usual source of care**				
Pharmacy	22 (19.8)	68 (58.1)	20 (18.2)	110 (32.5)
Health centre/clinic	29 (26.1)	2 (1.7)	17 (15.6)	48 (14.2)
Hospital	60 (54.1)	47 (40.2)	73 (66.4)	180 (53.3)
**Sex**				
Female	81 (62.3)	0	213 (99.1)	294 (59.4)
Male	49 (37.7)	150 (100.0)	2 (0.9)	201 (40.6)
** Age, M [SD]**	46.9 [13.9]	47 [11.6]	39.5 [8.2]	43.7 [11.5]
**Ethnic group**				
Ewe	6 (4.6)	37 (24.7)	70 (32.6)	113 (22.8)
Ga/Dangme	0 (0.0)	19 (12.7)	37 (17.2)	56 (11.3)
Akan	64 (49.2)	75 (50.0)	92 (42.8)	231 (46.7)
Busanga	24 (18.5)	0	0	24 (4.9)
Other	36 (27.7)	19 (12.7)	16 (7.4)	71 (14.3)
**Location of residence**				
Rural	127 (97.7)	7 (4.7)	8 (3.7)	142 (28.7)
Urban	3 (2.3)	143 (95.3)	207 (96.3)	353(71.3)
**Marital status**				
Single	3 (2.3)	20 (13.3)	52 (24.2)	75 (15.2)
Married/Co-habitation	116 (89.2)	120 (80.0)	139 (64.7)	375 (75.8)
Divorced/Separated/Widowed	11 (8.5)	10 (6.7)	24 (11.2)	45 (9.1)
** Household size, M [SD]**	7.9 [3.72]	5.0 [2.9]	4.7 [2.2]	5.6 [3.2]
**Highest education level**				
None	53 (41.1)	8 (5.3)	8 (4.3)	70 (14.2)
Primary	15 (11.6)	11 (7.3)	21 (11.3)	49 (9.9)
Middle school/JSS/JHS	53 (41.1)	99 (66.0)	113 (60.8)	286 (57.9)
SSS/SHS/Technical/Vocational	6 (4.7)	24 (16.0)	42 (22.6)	77 (15.6)
Tertiary	2 (1.6)	8 (5.3)	2 (1.1)	12 (2.4)
**Occupational status**				
Self-employed	119 (91.5)	69 (46.0)	210 (97.7)	398 (80.4)
Permanent Worker	1 (0.8)	33 (22.0)	5 (2.3)	39 (7.9)
Casual worker	10 (7.7)	48 (32.0)	0 (0.0)	58 (11.7)
** Median monthly income (GH¢)**	300	600	400	600

*M* Mean, *SD* Standard Deviation, *JHS* Junior High School, *SSS* Senior Secondary School, *SHS* Senior High School

## Effect of social capital on NHIS enrolment

The multinomial regression model demonstrated that not all the social capital variables showed a significant effect on enrolment in the NHIS; although effect of social capital variables was significantly positive overall (χ^2^ (4) = 45.64, *p* < 0.0001). Collective action was positively and significantly (*p* = 0.028) associated with enrolment in the NHIS (Table 2). Occupational group members who had attained Senior Secondary School (SSS)/Senior High School (SHS) or vocational/technical graduate also showed positive association with enrolment in the NHIS (*p* = 0.028). In addition, occupational group members with larger households had a significant positive relationship (*p* = 0.033) with enrolment in the NHIS.

**Table 2:** Multinomial logic regression model estimates for NHIS enrolment

Variable	Currently enrolled	Previously enrolled
	**Coef**	**Coef**
**Social capital**		
Diversity of membership	-8.45*** (3.16)	-3.12 (3.54)
Democratic functioning	-0.53 (1.36)	0.25 (1.05)
Bonding (within group interaction)	-1.09*** (0.26)	-0.64 (0.49)
Bridging (outside group interaction)	1.98 (1.26)	1.34 (1.04)
Trust in occupational and linguistic group	0.14 (0.08)	0.11*** (0.04)
Solidarity	-0.10 (0.13)	-0.11 (0.08)
Trust in NHIS and HCP	0.07 (0.21)	-0.11 (0.08)
Trust in transactions	-0.09 (0.17)	-0.08 (0.07)
Collective action	1.89** (0.86)	0.13 (0.87)
**Sex**		
Female^a^		
Male	-1.55** (0.71)	-0.93 (0.60)
Age	0.02 (0.03)	0.02 (0.01)
**Marital status**		
Married^a^		
Single	0.31 (0.32)	0.40*** (0.13)
Divorced/separated/widowed	-0.04 (0.80)	0.20 (0.77)
**Household size**	0.08** (0.04)	0.07*** (0.01)
**Ethnicity**		
Akan^a^		
Ewe	0.46 (0.43)	0.35 (0.32)
Ga/Dangme	-0.22*** (0.09)	-0.72*** (0.18)
Busanga	-0.57 (1.34)	-0.62 (0.79)
Other	0.45 (0.38)	-0.26 (0.32)
**Highest education level**		
None^a^		
Primary	-1.13 (0.89)	-0.03 (0.44)
Middle school/JSS/JHS	-0.30*** (0.07)	0.65*** (0.20)
Secondary/SSS/SHS/Vocational/Technical	0.21** (0.10)	1.19*** (0.22)
Tertiary	1.05* (0.57)	1.26*** (0.26)
**Occupational sector**		
Industry/commercial^a^		
Service	-1.29* (0.75)	-0.46 (0.60)
Agriculture	-1.32 (0.85)	-0.10 (0.71)
**Average monthly income**	0.00 (0.00)	0.00 (0.00)
**Self-reported health status**		
Very good^a^		
Good	0.23 (0.16)	-0.23 (0.20)
Fair	0.23 (0.49)	-0.08 (0.64)
Poor	-0.54 (1.46)	-0.28 (1.08)
**Usual source of care**		
Hospital^a^		
Clinic/Health centre	0.33 (0.30)	-0.29 (0.56)
Pharmacy	-1.47*** (0.44)	-0.53* (0.31)
**_cons**	6.42* (3.39)	1.87 (3.39)
Number of obs	455	
Pseudo R2	0.1602	
Log pseudolikelihood	-399.72426	
Hosmer–Lemeshow goodness-of-fittest	χ^2^ (16) = 19.112; *p* = 0.263	
Number of groups	10	
**Chi-squared statistic**	19.112	
**Prob > chi2**	0.263	

Robust standard errors are in parenthesis

Base or reference category for comparison was “Never enrolled” for the outcome variable

^***^p < 0.01

^**^p < 0.05

^*^p < 0.1

^a^Reference categories for the explanatory variables

Other social capital factors such as diversity of membership (*p* = 0.007) and bonding (within group interactions) (*p* < 0.001) were, however, negatively associated with enrolment in the NHIS. Occupational groups who had attained a middle school/Junior Secondary School (JSS)/Junior High School (JHS) level of education also had a significant negative relationship (*p* < 0.001) with enrolment in the NHIS. Likewise, being a male (*p* = 0.027) or belonging to the Ga/Dangme ethnic group (*p* = 0.009) was negatively associated with enrolment in the NHIS. Moreover, occupational group members who visited a private pharmacy for healthcare services, had a significantly negative association (*p* = 0.001) with enrolment in the NHIS.

The model further revealed that occupational groups with higher trust in their groups and other linguistic groups had a significantly positive association with (*p* = 0.005) dropout or previous enrolment in the NHIS. In addition, occupational group members with JSS/JHS (*p* = 0.001), SSS/JHS or vocational/technical (*p* < 0.001), and tertiary (*p* < 0.001) education had positive relationship with previous enrolment in the NHIS. The unmarried occupational group members also showed a significant positive relationship (*p* = 0.002) with previous enrolment in the NHIS. In addition, groups with lager household size had a significantly positive association (*p* < 0.001) with previous enrolment in the NHIS.

## Robustness of the multinomial regression model

The Homers-Lemeshow goodness-of-fit test showed a χ^2^ (16) = 19.112 and *p* = 0.263, indicating that the model is correctly specified. The “fitstat” test also showed *p* < 0.001, indicating that at least one of the regression coefficients in the model is not equal to zero.

## Marginal effects of occupational group on NHIS enrolment

**Fig. 1:**
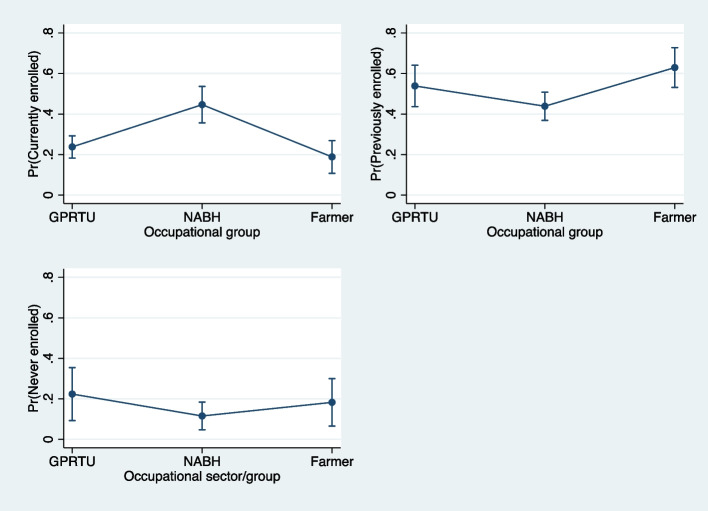
Predicted probabilities of enrolment with 95% CIs by occupational group

The probability of staying currently enrolled in the NHIS was 44.6% significantly higher (*p* < 0.001) for the beauticians and hairdressers’ group (NABH) than the private commercial road transport drivers (GPRTU) or the farmers, holding all other variables in the model constant at their means (Fig. 1). The predicted probability of dropping out of the NHIS or remaining previously enrolled was 62.9% significantly higher (*p* < 0.001) for the farmers than private commercial road transport drivers (GPRTU) or the beauticians and hairdressers’ group (NABH). However, the private commercial road transport drivers (GPRTU) had 22.3% significantly higher chance of never enrolling in the NHIS (*p* = 0.001), compared to the beauticians and hairdressers (NABH) or the farmers group.

## Discussion

Assessment of social determinants of NHIS enrolment shows that participation in the scheme is influenced by the level of social capital and type of informal sector occupational group. Occupational groups with higher level of collective action are more likely to enrol and stay active in the scheme. The plausible reason is that group members who work together for the benefit of the group are more likely to help each other in times of need. Such groups tend to have high solidarity, which serves as safety nets for the members. This finding, however, contradicts a study by Fenenga et al. [18], where collective action showed negative association with enrolment in the NHIS, although not significant.

Our study, however, reveals that occupational groups that are more diverse in membership and interact more frequently within their occupational groups (high level of bonding social capital) are less likely to enrol and remain active in the NHIS. It is evident that groups or associations with higher diversity of membership tend to yield more benefits to support themselves and the association through risk-sharing mechanisms [16, 46]. This finding indicates that relatively high level of social solidarity in the occupational groups may reduce participation in state-owned social programmes.

Findings of this study further show that occupational groups with higher trust in the group and other linguistic groups are more likely to drop out of the scheme, possibly due to benefits they derive from the group. The occupational groups help themselves in times of need including financial and healthcare needs; thus, they are not motivated enough to enrol in the scheme. Other study has also shown that groups or communities with high level of social solidarity and relatively low level of linkage with government institutions are less likely to participate in social and developmental programmes [47].

Analysis of the predicted probabilities of enrolling in the scheme reveals that the beauticians and hairdressers are significantly more likely to enrol and less likely to drop out, which suggests receipt of financial risk protection against their healthcare cost. Other studies have found that receipt of financial risk protection influences enrolment in health insurance programmes [48–51]. The free maternal healthcare services being offered by the NHIS might have also contributed to the high enrolment of beauticians and hairdressers in the scheme. This subpopulation is entirely women and majority are in the reproductive age group (15–49 years); therefore, they would find the scheme more attractive to their healthcare needs and participate in it.

The occupational groups in the agriculture sector (the farmers), on the other hand, are significantly less likely to enrol in the NHIS and more likely to drop out due to financial barrier and perceived good health status (Supplementary Table 5). This finding is consistent with a study which found that workers in the professional sector and sales or retail sector are more likely to enrol in the NHIS than those in the agriculture sector because of financial constraints [52]. Other studies in Ghana and elsewhere [19, 53–56] have also shown that poverty and affordability of insurance premiums are major barriers to enrolment [4, 5] particularly for poor households who are mostly farmers. Another reason for the high likelihood of dropout is that many of the farmers perceive themselves to be in good health; therefore, they do not need the health insurance, which corroborates other studies [19, 54] but contradicts one in Senegal, where perceived poor quality of health services was the most determinant of dropout in a community-based health insurance scheme [57]. The higher probability of dropping out of the NHIS for the farmers also contradicts a study in Indonesia, where households in the agricultural sector were more likely to continue to pay premium to stay insured [58].

Our study also shows that the private commercial road transport drivers are more likely to stay never enrolled and relatively more likely to drop out if enrolled, which can be attributed to the inconvenience associated with enrolment in the scheme and attitude of healthcare providers. Majority of the members cited long waiting hours and unfavourable office hours of work by the NHIS as the main reason for not enrolling or renewing membership in the scheme (Supplementary Table 5), consistent with other studies [12, 19, 59]. Our study also found that the private commercial road transport drivers demonstrated higher level of solidarity and transactions trust, which could explain their higher probability of never enrolling in the NHIS. Through risk-sharing mechanisms, groups with high level of solidarity help themselves in times of need, and this reduces participation in social intervention programmes [54, 60, 61] or yields little effect on enrolment in public health insurance programmes [20].

Other important individual level factors such as sex, level of education, ethnicity, and usual source of care also show significant relationships with active enrolment in the NHIS. Understandably, persons with basic level of education; the males; minority ethnic groups and persons who use pharmacy as their usual source of healthcare are less likely to enrol and stay active in the NHIS. These findings largely corroborate other studies conducted in Ghana [41, 53, 59, 62, 63] and elsewhere [4, 5].

Conversely, the unmarried (single, separated, widowed); families with large household sizes; and persons with basic level of education are more likely to drop out of the NHIS probably due to their limited knowledge of the scheme and financial constraints, as found in other studies [4, 64].

Findings of our study implies that social capital within informal sector occupational groups could be leveraged to improve group enrolment in the NHIS. The study further indicates that informal risk-sharing arrangements have the tendency to crowd out formal health insurance arrangements.

## Limitations

Social capital surveys solicit self-reported information relating to relationships in a group over a certain period, for example, in the past 12 months. This technique of collecting information often leads to recall bias since respondents hardly recollect events that happened to them a year ago. Nonetheless, questions were asked with emphasis on specific periods or special events to help the respondents recall. In addition, as with many group-based surveys, the sample size was relatively small for model stability in multinomial regression analysis, which uses a maximum likelihood estimation method. However, we clustered our observations into the three occupational groups and used them in the model equation to address the challenge of empty or small cells which causes model instability. We also acknowledge the potential effect of other variables (mediators and moderators), which were not included in this survey and recommend that future studies consider them to provide a deeper understanding of the relationship between social capital and enrolment in national health insurance schemes.

## Conclusions

Informal sector occupational groups that work collectively to address personal and group problems are more willing to adopt same group spirit to enrol in the NHIS. However, occupational groups that are more diverse, interact with themselves more frequently, and trust each other are less likely to enrol in the scheme. Notwithstanding these social determinants of NHIS enrolment, the predominantly female informal occupational groups are more likely to enrol in the scheme than the male dominated groups. Policy makers need to design group enrolment interventions targeting active informal occupational groups particularly the female dominated ones to improve enrolment in the NHIS towards attainment of UHC.

### Supplementary Information

**Supplementary Material 1.**

## Data Availability

The data for this study is deposited in Mendeley Data Repository with the Reserved 10.17632/cpj6pdr6zs.1. https://data.mendeley.com/datasets/cpj6pdr6zs.

## Abbreviations

GPRTU: Ghana Private Road Transport Union
HCP: Health Care Provider
NABH: National Association of Beauticians and Hairdressers
NHIA: National Health Insurance Authority
NHIS: National Health Insurance Scheme
PCA: Principal Component Analysis
SC: Social Capital
LMICs: Low-and Middle-Income Countries
OOP: Out-of-Pocket
CBHI: Community-based Health Insurance Scheme
UHC: Universal Health Coverage

## Footnotes

1 Median monthly income was reported rather than the mean because the income data was skewed.

2 US$ to GH¢ average rate for February 2019 was 5.21 (US$1 = GH¢5.21).
