# Supplementary material for: Effect of social capital on enrolment of informal sector occupational groups in the national health insurance scheme in Ghana: a cross-sectional survey
Source: BMC Health Serv Res. 2024 Apr 29;24:546. doi: 10.1186/s12913-024-11025-9 (PMC11059616; doi:10.1186/s12913-024-11025-9)
Supplement: Supplementary file 1 — Supplementary Material 1. [file 12913_2024_11025_MOESM1_ESM.pdf]

Supplementary Table 1: Key demographic and socio-economic variables for the study area

**Demographic and socioeconomic factors**	**Region**			
	**Greater Accra**	**Ashanti**	**Brong-Ahafo**	**Upper East**
**Demography**				
Population (2010 PHC)	4,010,054	4,780,380	2,310,983	1,046,545
Population growth rate (%)	3.1	2.7	2.3	1.2
Sex ratio (male/female*100)	93.6	94.0	98.2	93.8
Mean household size	2.6	2.9	3.6	4.3
**Education**				
Literacy rate (≥11 years)	80.5	71.1	55.5	37.3
**Main economic activity (occupation)**				
Service and sales workers	630,377	580,748	129,938	46,991
Skilled agriculture, forestry and fishery workers	44,961	303,560	464,657	175,981
Craft and related trade workers	409,921	336,957	120,574	35,118
Plant and machine union, and assemblers	98,250	86,109	24,938	4,037
Elementary occupations	80,865	80,020	63,598	22,876
**Employment status**				
Proportion of persons employed	67.7	62.5	72.6	58.7
Paid employee	539,441	476,817	112,400	44,190
Own account worker	917,831	992,658	521,160	248,828
Contributing family worker	48,441	79,802	160,267	7,942
Domestic worker	11,667	6,024	4,691	686
Casual worker	50,050	50,009	43,620	3,200
Apprentice	15,757	12,309	6,480	2,262
**Employment sector**				
Civil service worker	30,574	23,719	8,653	1,903
Public service worker	78,737	135,834	43,123	23,732
Private (including cooperatives)	1,464,132	1,496,589	797,049	283,496

**Poverty incidence 2012/13 (%)**	5.6	14.8	27.9	44.4
**Health**				
Number of persons enrolled in NHIS	1,410,206	1,968,878	1,325,075	713,334
Number of informal sector workers enrolled	506,985	557,303	396,403	138,122
Population to doctor ratio (year 2017)	3,052	6,888	10,059	26,484
**Health facilities (year 2017)**				
Hospitals	107	153	39	10
Health centres	40	164	131	55
Clinics & Polyclinics	336	150	78	46
Maternity homes	104	69	39	2
CHPS	498	1,122	665	255

PHC-Population and Housing Census; CHPS: Community-based Health Planning and Services. **Source**: [23–29]

Supplementary Table 2: Principal factor components and eigenvalues

Factor	Eigenvalue	Difference	Proportion	Cumulative
Factor1	3.63031	1.68232	0.3025	0.3025
Factor2	1.94799	0.78414	0.1623	0.4649
Factor3	1.16385	0.03831	0.0970	0.5618
Factor4	1.12554	0.23284	0.0938	0.6556
Factor5	0.89270	0.16915	0.0744	0.7300
Factor6	0.72355	0.16031	0.0603	0.7903
Factor7	0.56324	0.01087	0.0469	0.8373
Factor8	0.55238	0.07937	0.0460	0.8833
Factor9	0.47301	0.09423	0.0394	0.9227
Factor10	0.37878	0.06345	0.0316	0.9543
Factor11	0.31533	0.08204	0.0263	0.9806
Factor12	0.23329	.	0.0194	1.0000

LR test: independent vs. saturated: $\chi^2(66) = 1802.49$ Prob>$\chi^2 = 0.0000$

Supplementary Table 3: Rotated factor loadings (pattern matrix) and unique variances sorted

Variable	Factor1	Factor2	Factor3	Factor4	Uniqueness	KMO	Cronbach's α
q1_30_6	**0.7070**	-0.1535	0.3805	-0.0910	0.3235	0.8982	0.7494
q1_30_7	**0.6856**	-0.0973	0.3928	-0.0858	0.3588	0.7000	
q1_30_5	**0.6585**	0.2566	0.0800	0.0802	0.4877	0.8344	
q1_30_1	**0.6153**	0.2974	0.2228	-0.1319	0.4659	0.5661	
q1_30_3	**0.5884**	0.4767	-0.1174	0.0769	0.4069	0.8667	
q1_30_11	0.0277	**0.8844**	0.0608	-0.0334	0.2123	0.7358	0.7792
q1_30_12	-0.0104	**0.8721**	0.1667	0.0217	0.2111	0.7490	
q1_30_10	0.4580	**0.6261**	-0.0681	-0.0365	0.3922	0.7191	
q1_30_9	0.1564	0.0957	**0.8551**	-0.0436	0.2333	0.7216	0.7619
q1_30_8	0.1446	0.1136	**0.8390**	-0.0128	0.2621	0.8428	
q1_30_2	-0.1761	-0.1034	0.0262	**0.7618**	0.3772	0.6659	0.2966
q1_30_4	0.0935	0.0830	-0.1081	**0.7560**	0.4012	0.6661	
Overall						0.7601	0.7526

KMO: Kaiser-Meyer-Olkin measure of sampling adequacy

Supplementary Table 4: Average marginal effect estimates for the covariates for each outcome alternative (n=455)

Variable	Delta-method					
	**dy/dx**	**Std. Err.**	**z**	**P>z**	**[95% Conf.**	**Interval]**
**Social capital**						
Diversity membership						
_predict						
1	-1.071372	.2195127	-4.88	0.000	-1.501609	-.6411348
2	.4500469	.4353053	1.03	0.301	-.4031359	1.30323
3	.621325	.4287129	1.45	0.147	-.2189368	1.461587
Democratic functioning						
_predict						
1	-.1285419	.1221313	-1.05	0.293	-.367915	.1108311

2	.1296619	.1029204	1.26	0.208	-.0720585	.3313822
3	-.0011199	.1445501	-0.01	0.994	-.2844329	.2821931
Bonding (within group interaction)						
_predict						
1	-.1053721	.063518	-1.66	0.097	-.229865	.0191209
2	.0041723	.0996045	0.04	0.967	-.1910489	.1993936
3	.1011997	.0478679	2.11	0.035	.0073803	.1950192
Bridging (outside group interaction)						
_predict						
1	.1665733	.1047781	1.59	0.112	-.0387879	.3719346
2	.0341124	.103157	0.33	0.741	-.1680717	.2362964
3	-.2006857	.1374115	-1.46	0.144	-.4700073	.0686359
Trust in occupational and linguistic group						
_predict						
1	.0100139	.0131679	0.76	0.447	-.0157947	.0358226
2	.0051661	.0102483	0.50	0.614	-.0149202	.0252524
3	-.01518	.0058668	-2.59	0.010	-.0266788	-.0036812
Solidarity						
_predict						
1	-.0029425	.0165449	-0.18	0.859	-.0353699	.0294849
2	-.0111607	.0136413	-0.82	0.413	-.0378972	.0155759
3	.0141032	.0121226	1.16	0.245	-.0096568	.0378631
Trust in NHIS and HCP						
_predict						
1	.0281978	.0263701	1.07	0.285	-.0234866	.0798822

2	-.0353967	.0109694	-3.23	0.001	-.0568963	-.013897
3	.0071989	.0163269	0.44	0.659	-.0248012	.0391989
Trust in transactions						
_predict						
1	-.0049826	.0279654	-0.18	0.859	-.0597937	.0498286
2	-.006127	.0228359	-0.27	0.788	-.0508844	.0386305
3	.0111096	.010593	1.05	0.294	-.0096524	.0318715
Collective action						
_predict						
1	.3183683	.1521544	2.09	0.036	.0201512	.6165854
2	-.2310169	.1798192	-1.28	0.199	-.583456	.1214222
3	-.0873514	.0984945	-0.89	0.375	-.2803971	.1056944
**Sex**						
Female	(base outcome)					
Male						
_predict						
1	-.1565775	.050915	-3.08	0.002	-.256369	-.056786
2	-.0035386	.0479474	-0.07	0.941	-.0975138	.0904365
3	.1601161	.0892689	1.79	0.073	-.0148477	.33508
**Age**						
_predict						
1	-.0003779	.0044127	-0.09	0.932	-.0090266	.0082708
2	.0031543	.0026693	1.18	0.237	-.0020774	.008386
3	-.0027764	.0025615	-1.08	0.278	-.0077968	.0022439
**Marital status**						
Single						
_predict						

1	-.002388	.0522449	-0.05	0.964	-.104786	.10001
2	.0486471	.0430667	1.13	0.259	-.0357621	.1330563
3	-.0462591	.0197765	-2.34	0.019	-.0850203	-.0074979
married	(base outcome)					
Divorced/separated/widowed						
_predict						
1	-.0343996	.0387562	-0.89	0.375	-.1103604	.0415612
2	.0513861	.0711201	0.72	0.470	-.0880067	.190779
3	-.0169865	.0990099	-0.17	0.864	-.2110425	.1770694
**Household size**						
_predict						
1	.0045008	.006207	0.73	0.468	-.0076648	.0166663
2	.0051066	.0043748	1.17	0.243	-.0034678	.013681
3	-.0096074	.0021072	-4.56	0.000	-.0137375	-.0054773
**Ethnicity**						
Ewe						
_predict						
1	.032029	.027816	1.15	0.250	-.0224892	.0865473
2	.013291	.0119406	1.11	0.266	-.0101121	.0366942
3	-.0453201	.0395444	-1.15	0.252	-.1228257	.0321856
Ga/Dangme						
_predict						
1	.0543342	.0245611	2.21	0.027	.0061955	.102473
2	-.1347989	.039269	-3.43	0.001	-.2117647	-.0578331
3	.0804646	.0212251	3.79	0.000	.0388642	.1220651

Akan	(base outcome)					
Busanga						
_predict						
1	.1185339	.0278281	4.26	0.000	.0639919	.1730758
2	-.1218935	.0252543	-4.83	0.000	-.1713911	-.0723959
3	.0033596	.0410611	0.08	0.935	-.0771186	.0838379
Other						
_predict						
1	-.0182502	.1476114	-0.12	0.902	-.3075633	.2710629
2	-.0700819	.0896299	-0.78	0.434	-.2457534	.1055895
3	.0883322	.1469733	0.60	0.548	-.1997302	.3763945
**Highest education level**						
None	(base outcome)					
Primary						
_predict						
1	-.1963219	.107662	-1.82	0.068	-.4073355	.0146918
2	.1278617	.062683	2.04	0.041	.0050053	.250718
3	.0684602	.0822004	0.83	0.405	-.0926496	.22957
Middle school/JSS/JHS						
_predict						
1	-.1421356	.0260396	-5.46	0.000	-.1931723	-.0910989
2	.1821662	.0406848	4.48	0.000	.1024254	.2619069
3	-.0400306	.0164374	-2.44	0.015	-.0722472	-.007814
Secondary/SSS/SHS/vocational						
_predict						

1	-.1307849	.0231626	-5.65	0.000	-.1761828	-.0853869
2	.2318463	.0378924	6.12	0.000	.1575786	.306114
3	-.1010614	.0203275	-4.97	0.000	-.1409026	-.0612202
Tertiary						
_predict						
1	.017628	.0942723	0.19	0.852	-.1671424	.2023983
2	.1119337	.0859966	1.30	0.193	-.0566165	.280484
3	-.1295617	.0250245	-5.18	0.000	-.1786088	-.0805145
**Occupational sector**						
Service						
_predict						
1	-.173754	.0620737	-2.80	0.005	-.2954161	-.0520918
2	.0744549	.0401344	1.86	0.064	-.0042072	.1531169
3	.0992991	.091887	1.08	0.280	-.0807961	.2793943
Industry/commercial	(base outcome)					
Agriculture						
_predict						
1	-.2192247	.0765323	-2.86	0.004	-.3692251	-.0692242
2	.1590061	.0770233	2.06	0.039	.0080432	.309969
3	.0602186	.0940077	0.64	0.522	-.1240332	.2444704
**Average monthly income**						
_predict						
1	4.31e-06	.0000367	0.12	0.906	-.0000675	.0000762
2	1.98e-07	.0000413	0.00	0.996	-.0000807	.0000811
3	-4.51e-06	8.61e-06	-0.52	0.600	-.0000214	.0000124
**Self-reported health status**						

Very good	(base outcome)					
Good						
_predict						
1	.0721541	.0428033	1.69	0.092	-.0117387	.156047
2	-.0834921	.0522098	-1.60	0.110	-.1858215	.0188373
3	.0113379	.0190328	0.60	0.551	-.0259657	.0486416
Fair						
_predict						
1	.0517758	.0096334	5.37	0.000	.0328946	.0706569
2	-.0502598	.0774851	-0.65	0.517	-.2021278	.1016083
3	-.001516	.0754695	-0.02	0.984	-.1494335	.1464015
Poor						
_predict						
1	-.0530921	.2076275	-0.26	0.798	-.4600346	.3538503
2	.0041517	.2347888	0.02	0.986	-.4560259	.4643294
3	.0489404	.1545047	0.32	0.751	-.2538832	.351764
**Usual source of care**						
Hospital	(base outcome)					
Clinic/Health centre						
_predict						
1	.109002	.0837898	1.30	0.193	-.055223	.2732269
2	-.1140872	.1129781	-1.01	0.313	-.3355203	.1073459
3	.0050852	.0459513	0.11	0.912	-.0849777	.0951481
Pharmacy						
_predict						

1	-.1763348	.0748192	-2.36	0.018	-.3229777	-.0296918
2	.0595732	.0909635	0.65	0.513	-.118712	.2378583
3	.1167616	.0360793	3.24	0.001	.0460474	.1874758

Note: dy/dx for factor levels is the discrete change from the base level.

Outcome variable 1=Currently enrolled

Outcome variable 2=Previously enrolled

Outcome variable 3=Never enrolled

Supplementary Table 5: Reasons for not enrolling in the NHIS by occupational group (n=323)

**Variable**	**Farmers**	**Commercial road transport drivers**	**Beauticians and hairdressers**	**All**
	n (%)	n (%)	n (%)	n (%)
**Membership in other health insurance**				
I have other health insurance scheme	0	8 (6.0)	8 (7.2)	16 (5.0)
**Knowledge of NHIS**				
Never heard of NHIS	0	3 (2.3)	19 (17.1)	22 (6.8)
I have heard of NHIS but didn’t know benefits	0	18 (13.5)	22 (19.8)	40 (12.4)
Did not know how much to pay for premium	0	32 (24.1)	24 (21.6)	56 (17.3)
Did not know how to enrol and renew	0	28 (21.1)	19 (17.1)	47 (14.6)
**Benefits of NHIS**				
NHIS does not cover health services needed	0	33 (24.8)	38 (34.2)	71 (22.0)
NHIS does not save healthcare cost	1 (1.3)	32 (24.1)	45 (40.5)	78 (24.2)

NHIS benefit is too low or not enough	1 (1.3)	29 (21.8)	45 (40.5)	75 (23.2)
**Affordability of contributions**				
Cannot afford the premium/registration/renewal fees	34 (43.0)	15 (11.3)	22 (19.8)	71 (22.0)
**Convenience of enrolment**				
Premium payment interval is too short (yearly)	1 (1.3)	13 (9.8)	30 (27.0)	44 (13.6)
Premium payment interval is too long (yearly)	1 (1.3)	12 (9.0)	24 (21.6)	37 (11.5)
Distance to NHIS office is too far	16 (20.3)	51 (38.4)	48 (43.2)	115 (35.6)
Working hours of NHIS is not good for me	10 (12.7)	63 (47.4)	31 (27.9)	104 (32.2)
Long waiting hours in NHIS office	4 (5.1)	94 (70.7)	71 (64.0)	169 (52.3)
**Need for healthcare services**				
Do not need health care/not sick/mostly healthy	19 (24.1)	37 (27.8)	47 (42.3)	103 (31.9)
Healthcare facilities are too far	0	30 (22.6)	27 (24.3)	57 (17.7)
Quality of health services are not good	1 (1.3)	23 (17.3)	37 (33.3)	61 (18.9)
Attitude of healthcare provider is not good	1 (1.3)	29 (21.8)	33 (29.7)	63 (19.5)
Waiting time for health services is too long	0	40 (30.1)	46 (41.4)	86 (26.6)
Working hours of health facilities are not good	0	19 (14.3)	24 (21.6)	43 (13.3)
**Attitude of household or community members towards NHIS**				

Household members not in favour of enrolling in the NHIS	16 (20.3)	22 (16.5)	15 (13.5)	53 (16.4)
Community members not in favour of enrolling in the NHIS	5 (6.3)	14 (10.5)	14 (12.6)	33 (10.2)
Other reason (e.g., lack of trust)	15 (19.0)	5 (3.8)	6 (5.4)	26 (8.1)
